# Clinical usefulness of the lymphocyte-to-monocyte ratio and aggregate index of systemic inflammation in patients with esophageal cancer: a retrospective cohort study

**DOI:** 10.1186/s12935-023-02856-3

**Published:** 2023-01-27

**Authors:** Hui-Ke Wang, Qian Wei, Ya-Lan Yang, Tai-Ying Lu, Yan Yan, Feng Wang

**Affiliations:** Department of Oncology, The First Affiliated Hospital of Zhengzhou University, No.50 Eastern Jianshe Road, Zhengzhou, 450052 Henan China

**Keywords:** Esophageal cancer, Inflammation, Nomogram, Prognosis, Restricted cubic spline

## Abstract

**Background:**

Multiple perioperative inflammatory markers are considered important factors affecting the long-term survival of esophageal cancer (EC) patients. Hematological parameters, whether single or combined, have high predictive value.

**Aim:**

To investigate the inflammatory status of patients with preoperative EC using blood inflammatory markers, and to establish and validate competing risk nomogram prediction models for overall survival (OS) and progression-free survival (PFS) in EC patients.

**Methods:**

A total of 508 EC patients who received radical surgery (RS) treatment in The First Affiliated Hospital of Zhengzhou University from August 5, 2013, to May 1, 2019, were enrolled and randomly divided into a training cohort (356 cases) and a validation cohort (152 cases). We performed least absolute shrinkage and selection operator (LASSO)-univariate Cox- multivariate Cox regression analyses to establish nomogram models. The index of concordance (C-index), time-dependent receiver operating characteristic (ROC) curves, time-dependent area under curve (AUC) and calibration curves were used to evaluate the discrimination and calibration of the nomograms, and decision curve analysis (DCA) was used to evaluate the net benefit of the nomograms. The relative integrated discrimination improvement (IDI) and net reclassification improvement (NRI) were calculated to evaluate the improvement in predictive accuracy of our new model compared with the AJCC staging system and another traditional model. Finally, the relationship between systemic inflammatory response markers and prognostic survival was explored according to risk plot, time-dependent AUC, Kaplan–Meier and restricted cubic spline (RCS).

**Results:**

Based on the multivariate analysis for overall survival (OS) in the training cohort, nomograms with 10 variables, including the aggregate index of systemic inflammation (AISI) and lymphocyte-to-monocyte ratio (LMR), were established. Time-dependent ROC, time-dependent AUC, calibration curves, and DCA showed that the 1-, 3-, and 5 year OS and PFS probabilities predicted by the nomograms were consistent with the actual observations. The C-index, NRI, and IDI of the nomograms showed better performance than the AJCC staging system and another prediction model. Moreover, risk plot, time-dependent AUC, and Kaplan–Meier showed that higher AISI scores and lower LMR were associated with poorer prognosis, and there was a nonlinear relationship between them and survival risk.

**Conclusion:**

AISI and LMR are easy to obtain, reproducible and minimally invasive prognostic tools that can be used as markers to guide the clinical treatment and prognosis of patients with EC.

## Introduction

EC is one of the most common cancers in the world [1], and esophageal squamous cell carcinoma (ESCC) is the main pathological type of EC in China. EC remains a fatal disease, as it is usually not detected until it has progressed to an advanced stage. Despite recent improvements in management and treatment, the prognosis of EC [2] remains poor. Due to the poor prognosis and high incidence of EC, it is particularly important to find effective evaluation factors in daily clinical practice that can provide a basis for formulating the optimal postoperative treatment plan.

Currently, numerous studies focus on analyzing tumor molecular levels but ignore the impact of systemic tumor factors on survival and prognosis. In 2011, Professor Weinberg et al*.* [3] proposed ten characteristics of tumor cells in the journal *Cell,* including (1) a self-sufficient growth signaling pathway, (2) insensitivity to growth signals, (3) avoidance of apoptosis, (4) unlimited replication potential, (5) continuous angiogenesis, (6) tissue infiltration and distant metastasis, (7) evasion of immune destruction, (8) promotion of the inflammatory state of tumors, (9) abnormal energy metabolism of tumor cells, and (10) genomic instability and mutation. Thus, we can infer the importance of the nutritional, inflammatory and immune status of cancer patients.

Inflammation is thought to be a hallmark feature that initiates and promotes tumorigenesis [4]. Inflammation at the site of a tumor is generally considered a local immune response, consisting of immune cells, inflammatory protein mediators, and cytokines, constructing the local tumor microenvironment. Tumor-derived cytokines and mediators are secreted into the systemic circulation to mediate communication with distant sites. Systemic inflammation, including circulating cytokines, circulating immune cells, and inflammation-related proteins, is critical for tumor metastasis and interacts with local tumor immune responses; it can be detected and often marks the presence and progression of cancer [5]. In recent years, a large number of clinical trials have also reported systemic inflammatory indicators, such as the ratio of neutrophil-to-lymphocyte ratio (NLR), platelet-to-lymphocyte ratio (PLR) and LMR, which are closely related to patient survival and prognosis of various malignant tumors [6].

Compared with other prognostic factors, the prognostic indices based on inflammation are easy to obtain from preoperative routine blood tests, which is convenient and feasible. However, previous reports mostly focus on the impact of single inflammatory markers on prognosis, providing limited information for clinical oncologists. In this study, composite blood inflammatory indicators, including NLR, PLR, neutrophil-to-monocyte ratio (NMR), LMR, systemic immune-inflammation index (SII), NLPR, systemic inflammation response index (SIRI), and AISI, were used to score the inflammatory state of preoperative EC patients and to explore the relationship with long-term survival prognosis. We innovatively proposed the above eight inflammatory markers, clinicopathological features, inflammatory cells and their prognostic value for EC. In conclusion, we hope this study helps to guide the adjuvant treatment in patients with EC by studying simple and reliable relevant factors that are easy to obtain clinically.

## Materials and methods

### Study population

This retrospective study included 508 patients with EC who underwent RS at The First Affiliated Hospital of Zhengzhou University from August 5, 2013, to May 15, 2019.

The inclusion criteria were as follows: (1) patients with primary EC found by preoperative gastroscopy and pathological examination who underwent preoperative computed tomography (CT) or magnetic resonance imaging (MRI) and gastroscopy; (2) RS was performed in our hospital; (3) no significant abnormalities were found in liver, kidney, lung, brain, heart and bone marrow upon admission, and the laboratory tests were complete 1 week before surgery, including routine blood, liver and kidney function, electrolytes, blood biochemistry, hemagglutination, infectious diseases, routine urine, routine stool, tumor markers, etc*.*; (4) complete hospitalization records; (5) patients in whom the expected survival was more than 3 months, and the follow-up was estimated to last at least 12 months and who had complete postoperative follow-up data.

Relevant patients were selected according to the inclusion criteria. The clinicopathological stages were classified in accordance with the eighth edition of the AJCC staging system. Tumor classification was based on WHO classification guidelines. Tumor size was defined as the maximum diameter of gross pathology after RS. RS was defined as complete resection with a negative margin under a microscope. The primary endpoint of the whole study was OS, and the secondary outcome was PFS. OS was defined as the period from the date of randomization to the date of death or the last follow-up. PFS was defined as the period from the date of randomization to the earliest date of disease recurrence, namely, local recurrence or distant metastasis [7, 8].

All patients were followed up according to the standard postoperative schedule for EC as follows: the patients were followed-up every 3–4 months within 2 years with a chest CT, routine blood tests and tumor markers; after 2 years, the patients were followed-up every 6 months until 5 years after the operation. The patients were followed-up and rechecked with a chest, abdominal and pelvic enhanced CT and gastroscopy once a year. The last follow-up time was May 1, 2022, and the follow-up rate was 95.7%.

This study conformed to the principles of the Helsinki Declaration and relevant ethical requirements and was approved by the Ethics Committee of Scientific Research and Clinical Trials of the First Affiliated Hospital of Zhengzhou University (Approval Identifier: KY-2022-0361).

### Study variables

For all 508 EC patients, we recorded the following preoperative clinical data: age(y), sex, neoadjuvant therapy, comorbidity, treatment methods, histologic subtypes, histologic grade, T stage, N stage, M stage, TNM stage, tumor location, vascular invasion, nerve invasion, tumor size (cm), Hb, WBC, Neut, Mono, PDW, ALB, PA, LDL, CPR.

In addition, we calculated the inflammatory markers as follows: NLR = neutrophil/lymphocyte, PLR = platelet/neutrophil, NMR = neutrophil/monocyte, LMR = lymphocyte/monocyte, SII = (platelet × neutrophil)/lymphocyte, NLPR = neutrophil/(lymphocyte × platelet), SIRI = (neutrophil × monocyte)/lymphocyte, and AISI = (neutrophil × platelet × monocyte)/lymphocyte, based on the preoperative blood count.

### Study design and statistical analysis

**Fig. 1:**
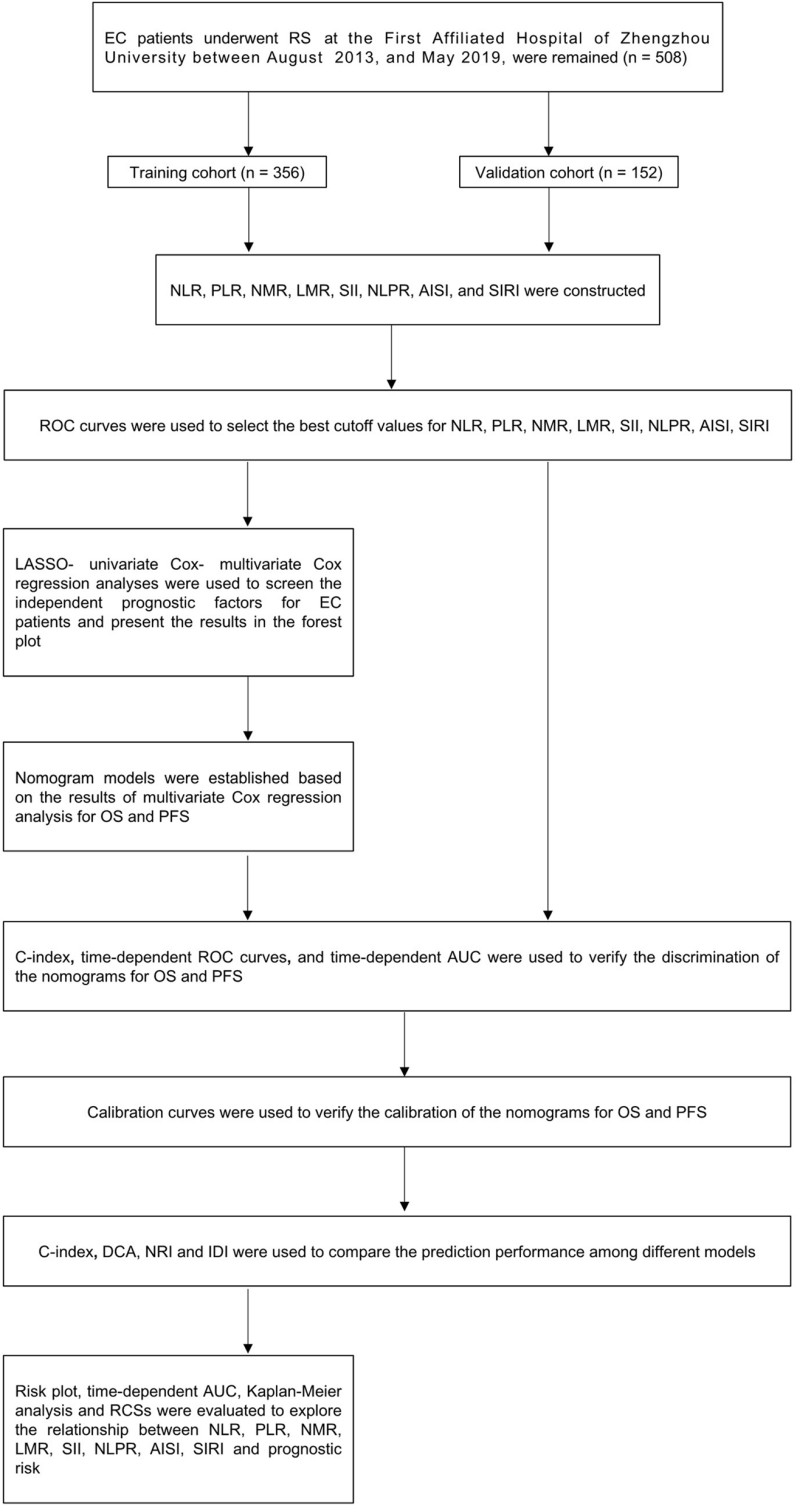
Flow chart of the study design

The study design is shown in Fig. 1. The patient cohort of The First Affiliated Hospital of Zhengzhou University was used as the training cohort to construct nomogram models including LMR and AISI, and the internal validation of the model was carried out in the training cohort.

Using ROC curves, the maximum sensitivity and specificity were calculated, the cutoff values of the parameters were selected, and the patients were divided into groups with high and low levels according to each cutoff value. All patients were randomly divided into a training cohort and a validation cohort at a ratio of 7:3. To compare variables between the training and validation cohorts, we used the chi-square test.

The variables selected by LASSO and those with *P* < 0.10 in univariate COX analysis were included in the multivariate analysis for mixed, forward, backward and stepwise regression analysis. The variable combination with the smallest akaike information criterion (AIC) value was selected by analysis of variance (ANOVA), and the variables with *P* < 0.05 were eligible to be used to generate the nomograms.

Subsequently, internal validation of the nomograms and comparison between the models were carried out. The C-index, time-dependent ROC and time-dependent AUC were used to evaluate the discrimination ability. If the C-index and AUC values were between 0.5 and 0.6, between 0.6 and 0.7, or greater than 0.8, the prediction performance of the model was considered to be poor, fair or good, respectively. Calibration curves were used to evaluate the calibration ability, and the bootstrap method was used to test the internal validity of the prediction models. Additionally, DCA was applied to assess the net benefit of the nomograms in a clinical context. The clinical benefit and utility of the NRI and IDI in evaluating the nomogram models compared with AJCC staging system and another prediction model further demonstrated the superiority of our models. If NRI and IDI > 0, it indicated positive improvement, indicating that the predictive ability of the new model was improved compared with that of the old model. If NRI and IDI < 0, it indicates a negative improvement, indicating a decrease in the predictive power of the new model.

A risk plot was used to show the differences in the distribution of population proportion, survival time and research indicators between the high- and low-risk groups. Time-dependent AUC was used to assess the effect of variables that changed over time on survival and recurrence. Survival curves were generated with the Kaplan‒Meier method, and the log-rank test was applied to compare the OS and PFS between different groups. We also flexibly modeled the nonlinear relationships between the inflammatory marks and the HR of the OS and PFS using RCSs at four nodes located at the 5th, 35th, 65th, and 95th percentiles.

All statistical analyses were performed with SPSS 26.0 and RStudio 4.2.1 software. The hazard ratio (HR) and the 95% confidence interval (CI) were employed to quantify the correlations between predictors and survival rate, with *P* < 0.05 indicating statistical significance. The significance level was α = 0.05.

## Results

### Patient characteristics and optimal cut-off values for the biomarkers

According to the inclusion criteria, 508 EC patients were finally included. The included data were randomly divided into a training cohort (n = 356) and a validation cohort (n = 152) at a ratio of 7:3.

The ROC curve was used to analyze the optimal threshold for predicting death with the highest sensitivity and specificity. The optimal cut-off values of NLR, PLR, NMR, LMR, SII, NLPR, SIRI, and AISI are 1.685, 83.590, 6.959, 4.724, 334.165, 0.006, 0.901, and 178.055, respectively, and the corresponding sensitivity and specificity are shown in Table 1. We divided the 508 patients into two groups based on the cutoff values calculated for the levels of inflammatory markers and the laboratory parameters.

**Table 1:** Diagnostic value of the parameters

Parameters	Cutoff value	Sensitivity	Specificity	AUC	95% CI	*P* value
NLR	1.685	0.587	1.000	0.814	0.771–0.856	< 0.001
PLR	83.590	0.629	0.925	0.789	0.745–0.834	< 0.001
NMR	6.959	0.675	0.660	0.642	0.552–0.731	0.001
LMR	4.724	1.000	0.602	0.816	0.774–0.857	< 0.001
SII	334.165	0.580	0.981	0.807	0.764–8.849	< 0.001
NLPR	0.006	0.723	0.830	0.812	0.767–0.857	< 0.001
SIRI	0.901	0.585	1.000	0.817	0.775–0.860	< 0.001
AISI	178.055	0.574	1.000	0.813	0.770–0.856	< 0.001
RDW (%)	13.350	0.580	0.906	0.718	0.661–0.774	< 0.001
Hb (g/L)	126.500	0.868	0.442	0.646	0.587–0.704	0.001
WBC (× 10^9^/L)	6.305	0.378	0.736	0.526	0.449–0.603	0.531
Neut (× 10^9^/L)	3.205	0.622	0.943	0.803	0.758–0.848	< 0.001
Mono (× 10^9^/L)	0.405	0.629	0.868	0.795	0.746–0.844	< 0.001
ALB (g/L)	42.150	0.906	0.580	0.760	0.707–0.814	< 0.001
PA (mg/L)	208.500	0.591	0.830	0.716	0.652–0.780	< 0.001
LDL (mmol/L)	2.535	0.596	0.943	0.738	0.689–0.787	< 0.001
CRP (mg/L)	3.655	0.571	0.943	0.806	0.747–0.865	< 0.001

The demographic and baseline data of the studied cohort are shown in Table 2. The patients were mainly < 65 years old and male; had not received neoadjuvant therapy; did not exhibit comorbidities; were treated with chemotherapy; exhibited pathological grade II, Tis + T1 + T2 stage, N0 + N1 stage, M0 stage, 0 + I + II stage; displayed lower esophagus involvement; did not exhibit vascular invasion or nerve invasion; and had a tumor size ≥ 3 cm. In the whole population, there were no significant differences in demographic and clinical characteristics between the training cohort and validation cohort (*P* > 0.05) (Table 2).

**Table 2:** Demographic and clinical characteristics of patients with EC. (n = 508)

Variables	Validation cohort [cases (%)]	Training cohort [cases (%)]	Whole population [cases (%)]	*χ*^2^	*P* value
Age (y)				0.004	0.950
< 65	85 (55.9)	198 (55.6)	283 (55.7)		
≥ 65	67 (44.1)	158 (44.4)	225 (44.3)		
Sex				0.317	0.574
Female	53 (34.9)	115 (32.3)	168 (33.1)		
Male	99 (65.1)	241 (67.7)	340 (66.9)		
Neoadjuvant therapy				0.745	0.388
Yes	112 (73.7)	275 (77.2)	387 (76.2)		
No	40 (26.3)	81 (22.8)	121 (23.8)		
Comorbidities				2.021	0.155
No	97 (63.8)	250 (70.2)	347 (68.3)		
Yes	55 (36.2)	106 (29.8)	161 (31.7)		
Treatment methods				3.741	0.154
Combination therapy	18 (11.8)	47 (13.2)	65 (12.8)		
Chemotherapy	88 (57.9)	230 (64.6)	318 (62.6)		
NC	46 (30.3)	79 (22.2)	125 (24.6)		
Histologic subtypes				0.029	0.865
ESCC	142 (93.4)	334 (93.8)	476 (93.7)		
Others	10 (6.6)	22 (6.2)	32 (6.3)		
Histological grade				1.179	0.555
III	32 (21.1)	67 (18.8)	99 (19.5)		
II	77 (50.7)	199 (55.9)	276 (54.3)		
I	43 (28.3)	90 (25.3)	133 (26.2)		
T stage				3.101	0.078
Tis + T1 + T2	94 (61.8)	190 (53.4)	284 (55.9)		
T3 + T4	58 (38.2)	166 (46.6)	224 (44.1)		
N stage				0.080	0.777
N0 + N1	127 (83.6)	301 (84.6)	428 (84.3)		
N2 + N3	25 (16.4)	55 (15.4)	80 (15.7)		
M stage				0.509	0.476
M0	151 (99.3)	351 (98.6)	502 (98.8)		
M1	1 (0.7)	5 (1.4)	6 (1.2)		
TNM stage				0.140	0.708
0 + I + II	86 (56.6)	195 (54.8)	281 (55.3)		
III + IV	66 (43.4)	161 (45.2)	227 (44.7)		
Tumor location				0.007	0.997
Upper	27 (17.8)	63 (17.7)	90 (17.7)		
Middle	53 (34.9)	123 (34.6)	176 (34.6)		
Lower	72 (47.4)	170 (47.8)	242 (47.6)		
Vascular invasion				0.342	0.559
No	112 (73.7)	271 (76.1)	383 (75.4)		
Yes	40 (26.3)	85 (23.9)	125 (24.6)		
Nerve invasion				2.127	0.145
No	119 (78.3)	298 (83.7)	417 (82.1)		
Yes	33 (21.7)	58 (16.3)	91 (17.9)		
Tumor size (cm)				0.318	0.573
< 3	66 (43.4)	145 (40.7)	211 (41.5)		
≥ 3	86 (56.6)	211 (59.3)	297 (58.5)		
NLR				0.364	0.546
< 1.685	69 (45.4)	172 (48.3)	241 (47.4)		
≥ 1.685	83 (54.6)	184 (51.7)	267 (52.6)		
PLR				0.399	0.527
< 83.590	62 (40.8)	156 (43.8)	218 (42.9)		
≥ 83.590	90 (59.2)	200 (56.2)	290 (57.1)		
NMR				1.588	0.208
< 6.959	61 (40.1)	122 (34.3)	183 (36.0)		
≥ 6.959	91 (59.9)	234 (65.7)	325 (64.0)		
LMR				1.367	0.242
< 4.724	88 (57.9)	186 (52.2)	274 (53.9)		
≥ 4.724	64 (42.1)	170 (47.8)	234 (46.1)		
SII				0.276	0.599
< 334.165	70 (46.1)	173 (48.6)	243 (47.8)		
≥ 334.165	82 (53.9)	183 (51.4)	265 (52.2)		
NLPR				0.521	0.470
< 0.006	50 (32.9)	129 (36.2)	179 (35.2)		
≥ 0.006	102 (67.1)	227 (63.8)	329 (64.8)		
SIRI				0.437	0.508
< 0.901	69 (45.4)	173 (48.6)	242 (47.6)		
≥ 0.901	83 (54.6)	183 (51.4)	266 (52.4)		
AISI				0.317	0.573
< 178.055	71 (46.7)	176 (49.4)	247 (48.6)		
≥ 178.055	81 (53.3)	180 (50.6)	261 (51.4)		
RDW (%)				0.238	0.626
< 13.350	69 (45.4)	170 (47.8)	239 (47.0)		
≥ 13.350	83 (54.6)	186 (52.2)	269 (53.0)		
Hb (g/L)				0.023	0.880
< 126.500	63 (41.4)	145 (40.7)	208 (40.9)		
≥ 126.500	89 (58.6)	211 (59.3)	300 (59.1)		
WBC (× 10^9^/L)				0.223	0.637
< 6.305	94 (61.8)	228 (64.0)	332 (63.4)		
≥ 6.305	58 (38.2)	128 (36.0)	186 (36.6)		
Neut (× 10^9^/L)				0.013	0.911
< 3.205	67 (44.1)	155 (43.5)	222 (43.7)		
≥ 3.205	85 (55.9)	201 (56.5)	286 (56.3)		
Mono (× 10^9^/L)				0.209	0.648
< 0.405	62 (40.8)	153 (43.0)	215 (42.3)		
≥ 0.405	90 (59.2)	203 (57.0)	293 (57.7)		
ALB (g/L)				0.083	0.773
< 42.150	79 (52.0)	190 (53.4)	269 (53.0)		
≥ 42.150	73 (48.0)	166 (46.6)	239 (47.0)		
PA (mg/L)				1.762	0.184
< 208.500	62 (40.8)	168 (47.2)	230 (45.3)		
≥ 208.500	90 (59.2)	188 (52.8)	278 (54.7)		
LDL (mmol/L)				0.951	0.330
< 2.535	65 (42.8)	169 (47.5)	234 (46.1)		
≥ 2.535	87 (57.2)	187 (52.5)	274 (53.9)		
CRP (mg/L)				3.257	0.071
< 3.655	64 (42.1)	181 (50.8)	245 (48.2)		
≥ 3.655	88 (57.9)	175 (49.2)	263 (51.8)		

### Screening for predictive factors

**Fig. 2:**
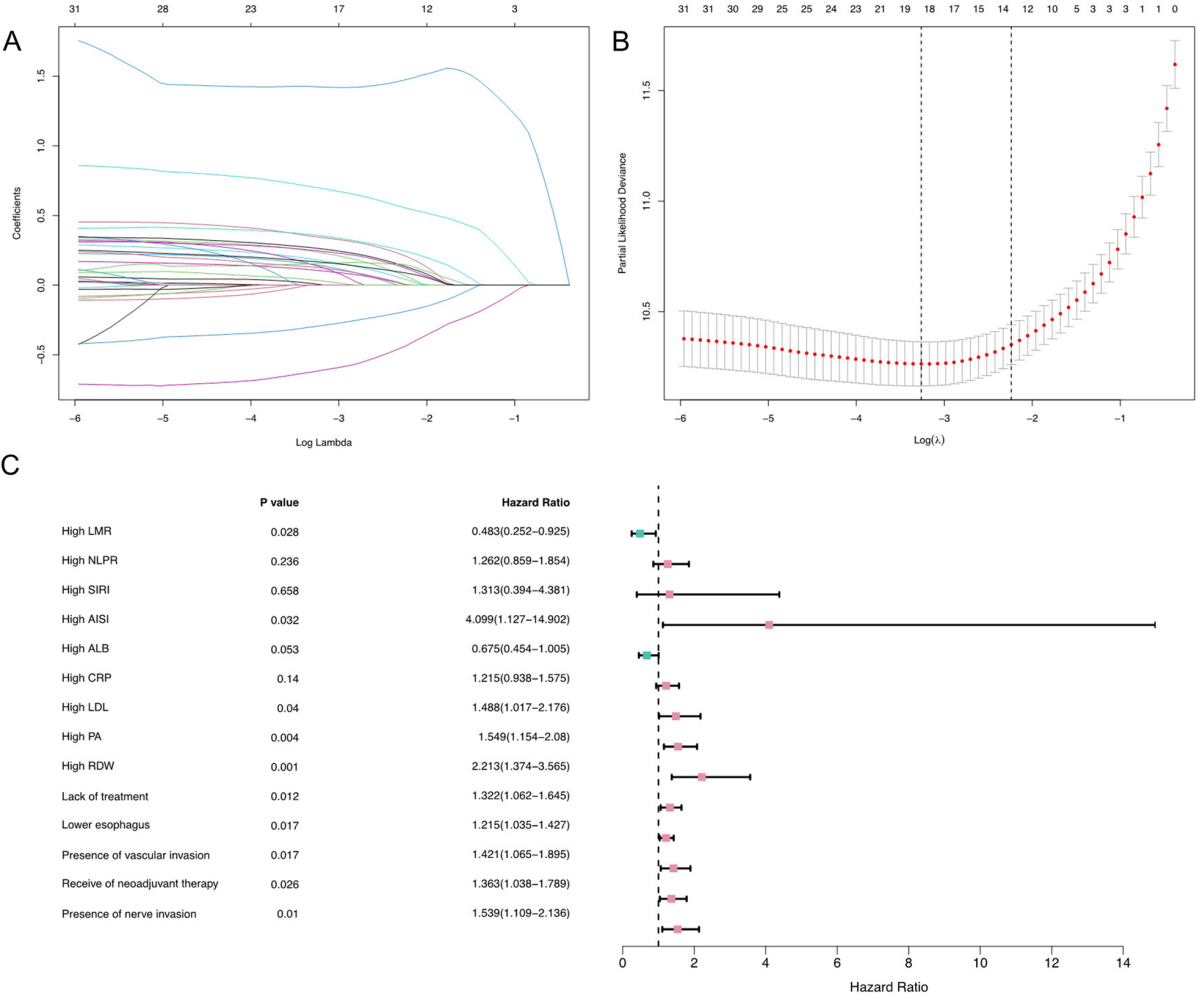
Determination of the number of factors by the LASSO- univariate Cox- multivariate Cox regression analyses. **A** LASSO coefficient profiles of the 32 survival-related factors in the training cohort. **B** Selection of the optimal parameter in the LASSO regression model; **C** Multivariate Cox regression analysis of EC based on LMR and AISI in the training cohort for OS

Through the LASSO Cox regression model (Fig. 2A, B), 14 indicators related to OS were screened and input into the univariate Cox regression model. Variables with *P* < 0.10 in the univariate analysis were included in the multivariate Cox regression, and the results showed that lack of treatment (*P* = 0.012, 95% CI 1.062–1.645), receival of neoadjuvant therapy (*P* = 0.026, 95% CI 1.038–1.789), presence of nerve invasion (*P* = 0.010, 95% CI 1.109–2.136), presence of vascular invasion (*P* = 0.017, 95% CI 1.065–1.895), location in lower esophagus (*P* = 0.017, 95% CI 1.035–1.427), high LDL (*P* = 0.040, 95% CI 1.017–2.176), high PA (*P* = 0.004, 95% CI 1.154–2.080), high RDW (*P* = 0.001, 95% CI 1.374–3.565), high AISI (*P* = 0.032, 95% CI 1.127–14.902), and high LMR (*P* = 0.028, 95% CI 0.252–0.925), may be independent predictors of EC patient OS (Table 3). In addition, we visualized the results of the above multivariate Cox regression in a forest plot (Fig. 2C).

**Table 3:** Univariate and multivariate Cox analyses on variables for the prediction of OS of EC patients

Variables	Univariate analysis			Multivariate analysis		
	HR	95% CI	*P* value	HR	95% CI	*P* value
High LMR	0.103	0.079–0.134	< 0.001	0.483	0.252–0.925	0.028
High NLPR	3.650	2.858–4.661	< 0.001	1.262	0.859–1.854	0.236
High SIRI	12.500	9.509–16.608	< 0.001	1.313	0.394–4.381	0.658
High AISI	21.739	15.704–30.631	< 0.001	4.099	1.127–14.902	0.032
High ALB	0.205	0.162–0.261	< 0.001	0.675	0.454–1.005	0.053
High CRP	2.639	2.095–3.320	< 0.001	1.215	0.938–1.575	0.140
High LDL	5.000	3.920–6.366	< 0.001	1.488	1.017–2.176	0.040
High PA	3.165	2.514–3.985	< 0.001	1.549	1.154–2.080	0.004
High RDW	7.463	5.795–9.592	< 0.001	2.213	1.374–3.565	0.001
Lack of treatment	1.425	1.165–1.744	0.001	1.322	1.062–1.645	0.012
Lower esophagus	1.248	1.076–1.449	0.004	1.215	1.035–1.427	0.017
Presence of vascular invasion	2.183	1.688–2.825	< 0.001	1.421	1.065–1.895	0.017
Receive of neoadjuvant therapy	1.887	1.458–2.440	< 0.001	1.363	1.038–1.789	0.026
Presence of nerve invasion	2.331	1.739–3.121	< 0.001	1.539	1.109–2.136	0.010

### Development of the nomogram

**Fig. 3:**
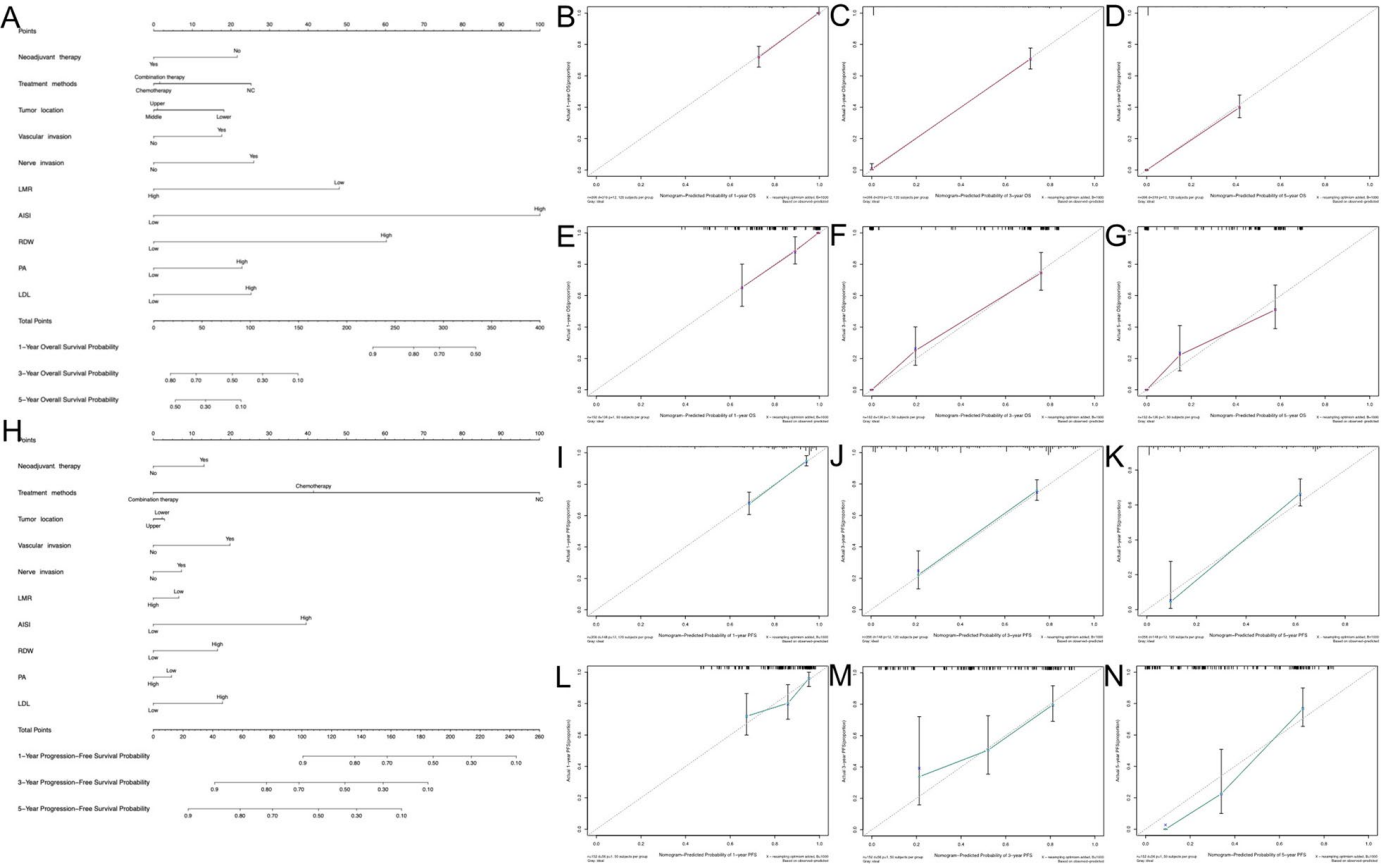
**A** Nomogram model of EC patients for predicting the 1 year, 3 year, and 5 year OS rates. To predict the 1 year, 3 year, and 5 year OS rates of EC patients, locate the patient’s LMR on the “LMR” axis. Draw a straight line up to the “point” axis to determine the points for “LMR”. Repeat the process for each of the remaining axes, drawing a straight line each time to the “point” axis. Add the points received from each variable and locate this point on the “total point” axis. A straight line is drawn down from the “total point” axis to the “1 year OS”, “3 year OS”, and “5 year OS” axis to determine the 1 year, 3 year, and 5 year OS rates of EC patients. Calibration curves of the nomogram in the training cohort **B** 1 year OS, **C** 3-year OS, **D** 5 year OS; Calibration curves of the nomogram in the validation cohort **E** 1-year OS, **F** 3 year OS, **G** 5-year OS. The X-axis represents the model − predicted survival, and the Y − axis represents actual survival. The bar represents 95% CI measured by Kaplan–Meier analysis, and the dotted line represents the ideal reference line. **H** Nomogram model of EC patients for predicting the 1 year, 3 year, and 5 year PFS rates. Calibration curves of the nomogram in the training cohort **I** 1 year PFS, **J** 3 year PFS, **K** 5 year PFS; Calibration curves of the nomogram in the validation cohort **L** 1 year PFS, **M** 3 year PFS, **N** 5-year PFS

Based on the independent risk factors screened by multivariate Cox regression, nomograms were generated to predict the risk of OS (Fig. 3A) and PFS (Fig. 3H) in EC patients. Each prognostic factor had a corresponding point, which enabled the risk of each factor to be transformed into a computable value. The total score was calculated by adding the scores of all prognostic factors, and a vertical line was drawn at the bottom of the probability line that corresponded to the probability that the patient would die. From the nomograms, we observed that LMR and AISI had the strongest prognostic value in predicting early mortality.

### Validation of the nomogram

The C-index, time-dependent ROC, and time-dependent AUC were used to evaluate the discrimination of the nomograms. The C-indices based on OS and PFS were 0.798 and 0.754 in the training cohort and 0.790 and 0.702 in the validation cohort, respectively (Table 4). Moreover, the calibration curves of the 1-, 3-, and 5 year OS and PFS probability nomograms for the training and validation cohorts showed that the observed results were consistent with the predicted results (Fig. 3).

**Table 4:** The Predictive Performance (C-Index, NRI and IDI) of Different Models for Predicting EC patients’ OS and PFS in the Training Cohort and Validation Cohort

Model	Author	Key Predictors of the Model	Index	Outcome	Training cohort			Validation cohort		
					Estimate	95% CI	*P* value	Estimate	95% CI	*P* value
Model proposed in this study	Huike Wang et al*.* 2022	A nomogram model including nerve invasion, vascular invasion, neoadjuvant therapy, tumor location, treatment methods, RDW, PA, LDL, AISI and LMR	C-index	OS	0.798	0.782 to 0.814	–	0.790	0.763 to 0.817	–
				PFS	0.754	0.717 to 0.791	–	0.702	0.677 to 0.727	–
Model A[38]	Xiang Lv et al*.* 2021	A nomogram model including age, TNM stage, T stage, N stage, LMR and NLR	C-index	OS	0.759	0.741 to 0.777	–	0.769	0.744 to 0.794	–
				PFS	0.704	0.663 to 0.745	–	0.619	0.542 to 0.695	–
			NRI	1-year OS	− 0.081	− 0.408 to − 0.137	–	− 0.102	− 0.625 to 0.159	–
				3-year OS	− 0.180	− 0.563 to − 0.009	–	− 0.195	− 0.581 to 0.234	–
				5-year OS	− 0.211	− 0.371 to 0.000	–	− 5.882	− 0.398 to − 0.316	–
				1-year PFS	− 0.186	− 0.306 to − 0.263	–	− 0.077	− 0.364 to − 0.246	–
				3-year PFS	− 0.240	− 0.482 to − 0.049	–	− 0.127	− 0.504 to 0.035	–
				5-year PFS	− 0.283	− 0.435 to − 0.089	–	− 0.305	− 0.516 to − 0.007	–
			IDI	1-year OS	− 0.046	− 0.104 to − 0.011	0.020	− 0.066	− 0.169 to 0.003	0.070
				3-year OS	− 0.054	− 0.096 to − 0.021	< 0.001	− 0.023	− 0.130 to − 0.036	0.338
				5-year OS	− 0.055	− 0.103 to − 0.016	< 0.001	− 0.023	− 0.128 to 0.046	0.448
				1-year PFS	− 0.039	− 0.120 to − 0.027	0.289	− 0.041	− 0.161 to − 0.048	0.338
				3-year PFS	− 0.117	− 0.187 to − 0.036	< 0.001	− 0.100	− 0.229 to − 0.010	0.030
				5-year PFS	− 0.148	− 0.243 to − 0.061	< 0.001	− 0.172	− 0.297 to − 0.022	0.030
The AJCC staging system		T stage, N stage and M stage	C-index	OS	0.542	0.511 to 0.573	–	0.544	0.519 to 0.569	–
				PFS	0.609	0.593 to 0.625	–	0.542	0.517 to 0.567	–
			NRI	1-year OS	− 0.417	− 0.575 to − 0.230	–	− 0.590	− 0.963 to − 0.297	–
				3-year OS	− 0.671	− 0.778 to − 0.567	–	− 0.694	− 0.878 to − 0.508	–
				5-year OS	− 0.211	− 0.371 to 0.000	–	− 0.311	− 0.666 to − 0.050	–
				1-year PFS	− 0.297	− 0.531 to − 0.062	–	− 0.469	− 0.797 to − 0.011	–
				3-year PFS	-0.497	− 0.902 to − 0.345	–	− 0.449	− 0.895 to − 0.110	–
				5-year PFS	-0.343	− 0.755 to − 0.211	–	− 0.369	− 1.160 to − 0.185	–
			IDI	1-year OS	− 0.173	− 0.247 to − 0.088	< 0.001	− 0.196	− 0.307 to − 0.126	< 0.001
				3-year OS	− 0.535	− 0.611 to − 0.448	< 0.001	− 0.788	− 0.868 to − 0.822	< 0.001
				5-year OS	− 0.314	− 0.375 to − 0.268	< 0.001	− 0.368	− 0.509 to − 0.265	< 0.001
				1-year PFS	− 0.136	− 0.220 to − 0.074	< 0.001	− 0.123	− 0.275 to − 0.035	< 0.001
				3-year PFS	− 0.348	− 0.437 to − 0.250	< 0.001	− 0.252	− 0.420 to − 0.123	< 0.001
				5-year PFS	− 0.349	− 0.431 to − 0.254	< 0.001	− 0.342	− 0.504 to − 0.172	0.010

**Fig. 4:**
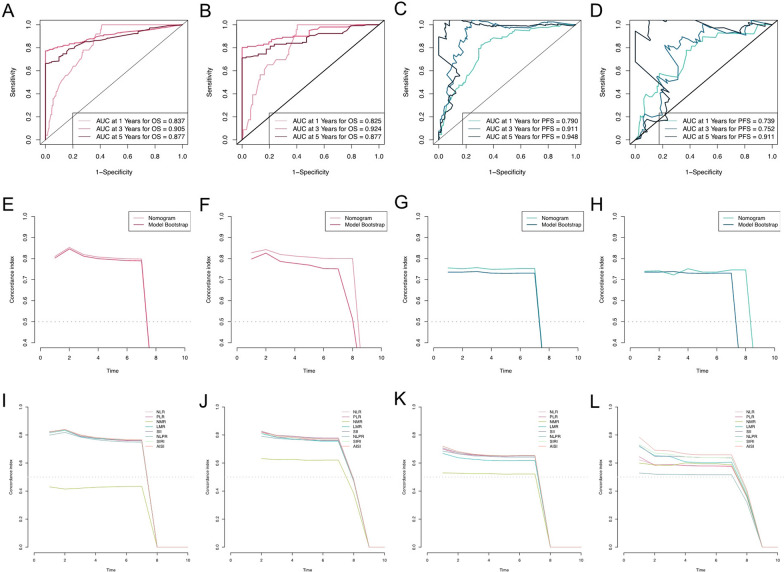
The prognostic performance of nomograms and the prognostic performance in patients with EC. The time-dependent ROC curves of the nomograms **A** for OS in the training cohort; **B** for OS in the validation cohort; **C** for PFS in the training cohort; **D** for PFS in the validation cohort. The time-dependent AUC curves of the nomograms **E** for OS in the training cohort; **F** for OS in the validation cohort; **G** for PFS in the training cohort; **H** for PFS in the validation cohort. The prognostic performance of the inflammatory marks in patients with EC. The time-dependent AUC curves of NLR, PLR, NMR, LMR, SII, NLPR, AISI, and SIRI **I** for OS in the training cohort; **J** for OS in the validation cohort; **K** for PFS in the training cohort; **L** for PFS in the validation cohort

The AUCs for predicting the 1-, 3-, and 5 year OS and PFS in the training cohort were 0.837, 0.905, and 0.877 (Fig. 4A) and 0.790, 0.911, and 0.948 (Fig. 4C), respectively, and those in the validation cohort were 0.825, 0.924, and 0.877 (Fig. 4B) and 0.739, 0.752, and 0.911 (Fig. 4D), respectively. The time-dependent AUCs for predicting OS and PFS over 7 years were all > 0.7, indicating the favorable discriminative ability of the nomograms (Fig. 4E–H).

### Comparison among different predictive models

**Fig. 5:**
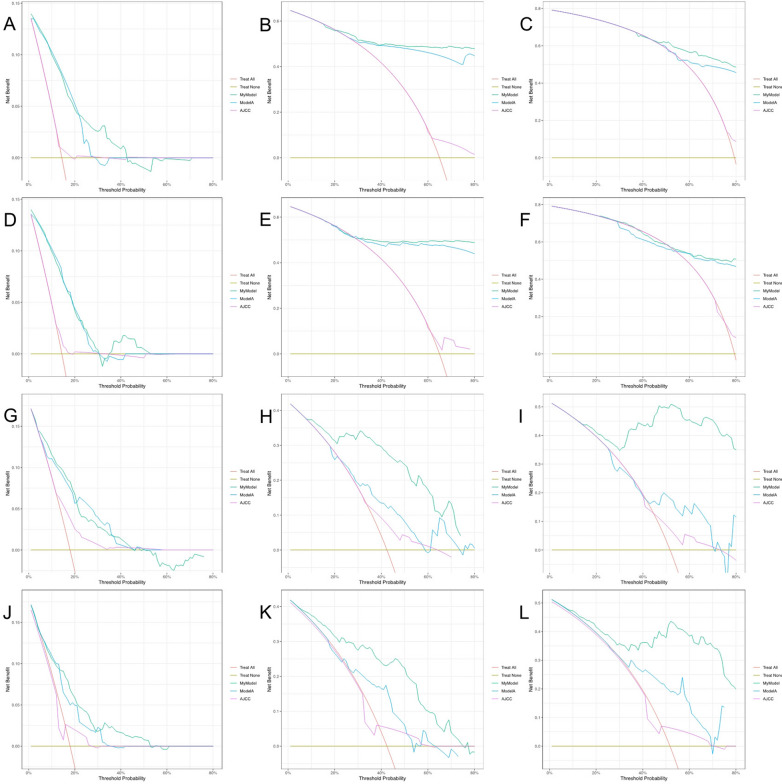
DCA curves of the nomograms and the AJCC staging system as well as another prediction model. The DCA curves were plotted based on **A** 1 year OS, **B** 3 year OS, **C** 5 year OS benefit in the training cohort; **D** 1 year OS, **E** 3 year OS, **F** 5 year OS benefit in the validation cohort; **G** 1 year PFS, **H** 3 year PFS, **I** 5 year PFS benefit in the training cohort; **J** 1 year PFS, **K** 3 year PFS, **L** 5 year PFS benefit in the validation cohort

DCA showed a significant improvement in the net benefit of the nomograms over the AJCC tumor staging system and other prediction models, with a wide range of threshold probabilities in both the training and validation cohorts (Fig. 5). This finding that the new nomogram models are more beneficial for clinical application in predicting individual survival outcomes than the AJCC staging system and another prediction model.

In the training cohort and validation cohort, we compared our models with the AJCC tumor staging system and other predictive models using IDI and NRI and compared their discriminative abilities using the C-index (Table 4). Their NRI and IDI were both less than 0, indicating a negative improvement, and their C- indices were smaller than those of our models. These results indicated that our models had better predictive power and discrimination ability than the AJCC staging system and another predictive model.

### The impact of the systemic inflammatory indices on the outcomes of interest

**Fig. 6:**
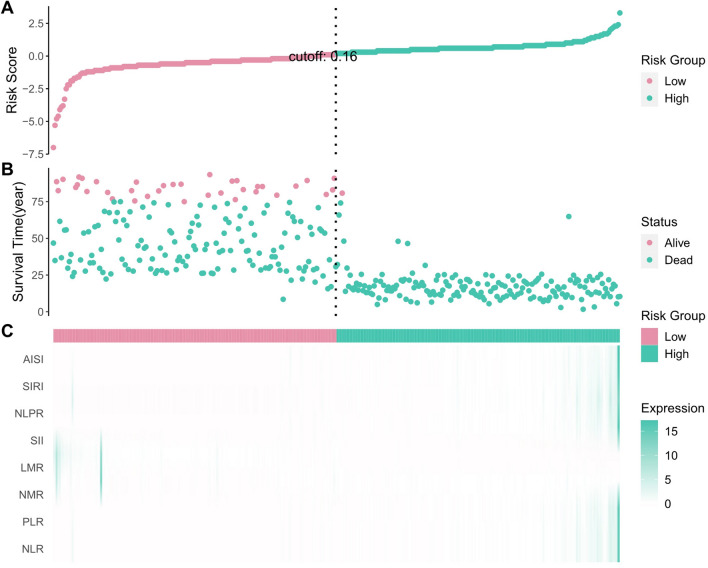
Relationship between the survival status/ risk score rank and survival time (year)/risk score rank. **A** The distribution of risk score; **B** The survival duration and status of EC patients; **C** A heatmap of NLR, PLR, NMR, LMR, SII, NLPR, AISI, and SIRI in the classifier

To further investigate the ability of the inflammatory indices to predict survival, we plotted scatter plots of indicator expression in different samples in addition to the corresponding OS (Fig. 6). For predicting OS over 7 years in both the training cohort and validation cohort, the AUCs achieved using the inflammatory indices ranged from 0.80 to 0.85 (except for NMR), indicating a reasonable assessment (Fig. 4I–L).

**Fig. 7:**
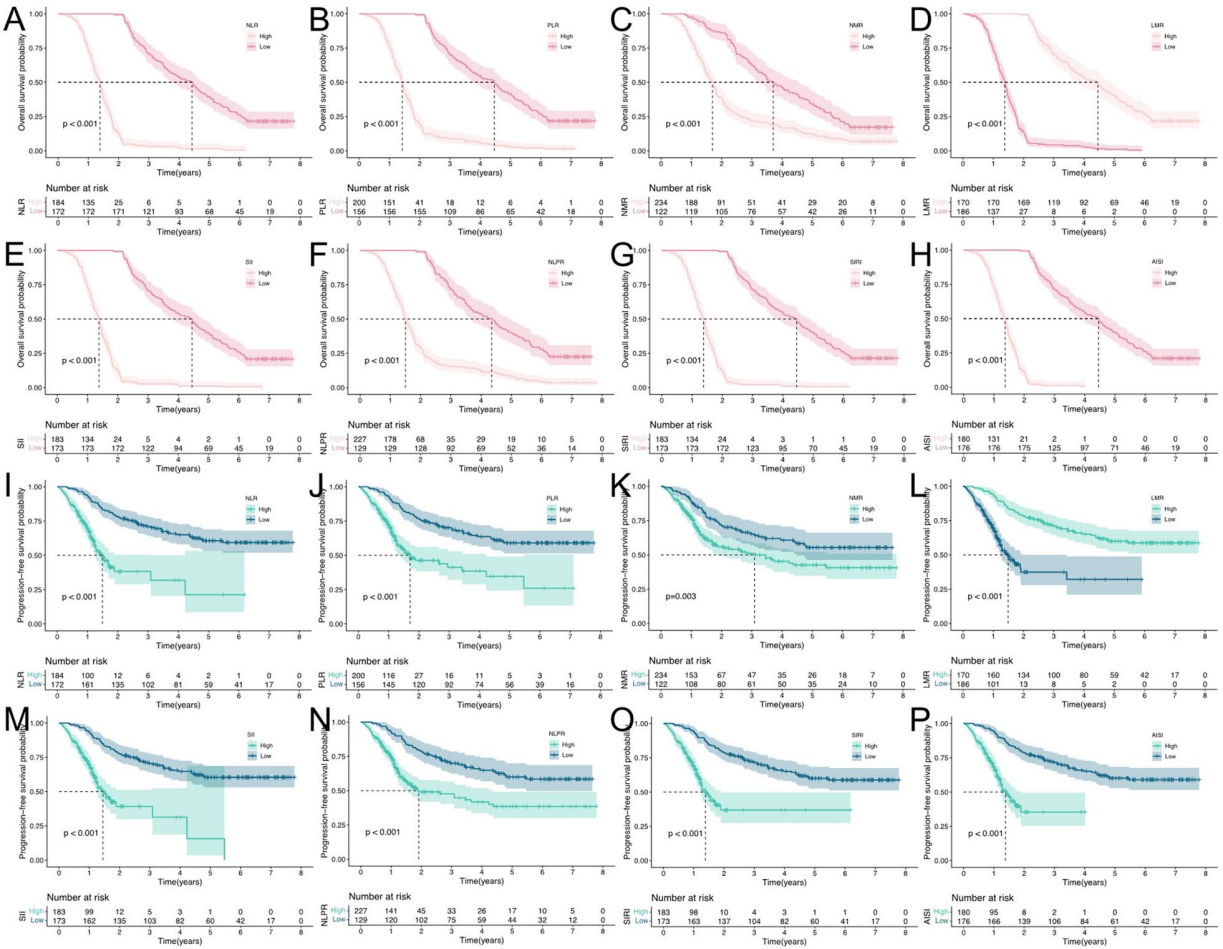
Kaplan–Meier curves for risk stratification. Kaplan–Meier plots for OS in the training cohort between **A** NLR, **B** PLR, **C** NMR, **D** LMR, **E** SII, **F** NLPR, **G** AISI, **H** SIRI risk score groups; for PFS in the training cohort between **I** NLR, **J** PLR, **K** NMR, **L** LMR, **M** SII, **N** NLPR, **O** AISI, **P** SIRI risk score groups

**Fig. 8:**
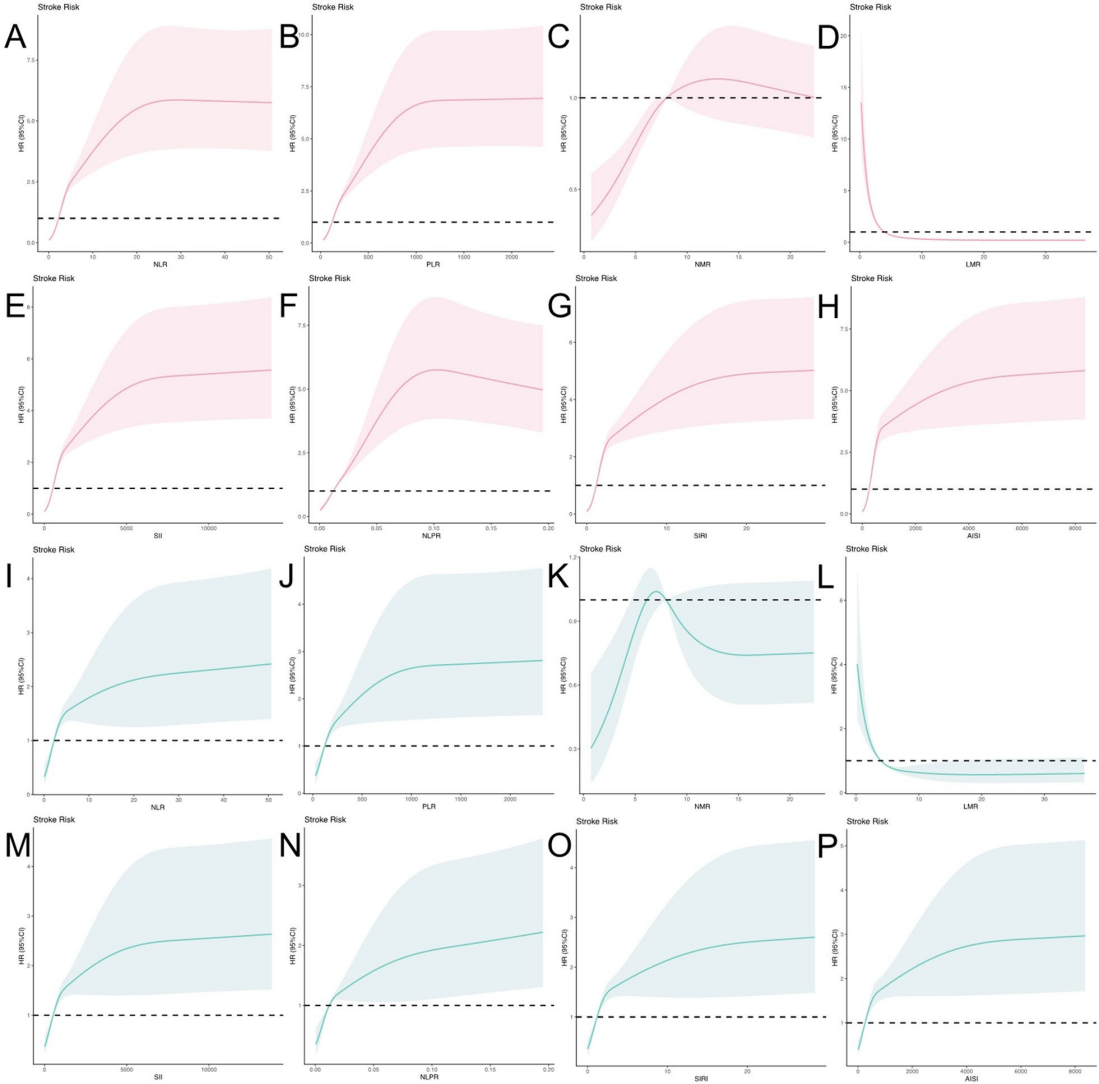
Association between NLR, PLR, NMR, LMR, SII, NLPR, AISI, SIR and HR for OS and PFS using RCS regression models in patients with EC. **A** NLR, **B** PLR, **C** NMR, **D** LMR, **E** SII, **F** NLPR, **G** AISI, **H** SIRI and OS in the training cohort; **I** NLR, **J** PLR, **K** NMR, **L** LMR, **M** SII, **N** NLPR, **O** AISI, **P** SIRI and PFS in the training cohort. (Unadjusted covariable)

Kaplan‒Meier curves and log-rank tests revealed that higher NLR, PLR, SII, NLPR, AISI, and SIRI and lower NMR and LMR were significantly associated with worse OS and PFS (all *P* < 0.05) (Fig. 7). Taking NLR, PLR, NMR, LMR, SII, NLPR, AISI, and SIRI as the variables of RCSs, as shown in Fig. 8, the nonlinear correlation *P* < 0.001 of the above variables indicated that there were nonlinear relationships between them and the HRs of OS and PFS.

## Discussion

Personalized therapy that can accurately predict the recurrence and prognosis of EC patients has been the direction of researchers’ efforts. In this study, we recruited 508 EC patients to investigate whether inflammatory markers such as NLR, PLR, NMR, LMR, SII, NLPR, SIRI, and AISI can be used as diagnostic and prognostic tools and to demonstrate that in addition to traditional clinicopathological parameters, LMR and AISI may also be independent predictors of EC recurrence.

To date, there is no consensus on which hematological biomarker is the best index to predict the prognosis of EC. NLR, PLR, LMR, NMR, SII, NLPR, AISI and SIRI are blood cell indices derived from CBCs, whose proportions may better represent the inflammatory state than individual ones [8]. Previous studies showed that NLR [9, 10], PLR [11], LMR [11, 12], SII [13, 14], and SIRI [15, 16] were associated with poor prognosis. In contrast, no studies have confirmed that NMR, NLPR and AISI are independent predictors of poor prognosis in patients with EC. To the best of our knowledge, this is the first report to evaluate the relationship between NMR, NLPR, and AISI and the prognosis of patients with EC. Our study showed significant changes in all blood-derived indicators, among which LMR and AISI were superior to the others, demonstrating that LMR and AISI were independent prognostic indicators. Compared with patients with lower AISI scores, the prognosis of patients with higher AISI scores was significantly worse. In addition, we also calculated the AUC and C-index of these biomarkers. LMR (AUC = 0.816) and AISI (AUC = 0.813) had large AUC values for OS, indicating that LMR and AISI are hematological biomarkers for predicting OS in EC patients who have undergone RS. These data provide an effective way for clinicians to identify high-risk EC patients with poor prognoses before treatment and further adjust individualized treatment plans or take pretreatment measures.

In recent years, there has been increasing evidence that inflammatory biomarkers are significantly associated with poor prognosis of EC. However, the exact mechanism remains unclear. Lymphocyte levels, neutrophil levels, monocyte levels, and platelet are thought to be involved in regulating inflammation [8]. First, lymphocyte levels is involved in immune regulation in the tumor microenvironment, which may establish an immune response to tumor cells in humans [17]. Thus, a low lymphocyte levels count correlates with immunosuppressive status, which provides a favorable microenvironment for tumor proliferation and migration [18]. Second, neutrophil levels counts are increased both in the tumor microenvironment and throughout the body and are often associated with poor outcomes in solid cancer patients [19]. As an inflammatory response, it suppresses the immune system by inhibiting the cytolytic activity of immune cells such as lymphocyte levels and activating T cells and natural killer cells [20]. Endothelial and parenchymal cells can also be activated to enhance circulating tumor cell adhesion and promote distant metastasis [21]. Third, circulating monocytes have been found to promote tumor growth and help tumor cells evade immune surveillance [22, 23]. In addition, it was reported that tumor-associated macrophages (TAM) derived from circulating monocytes can penetrate the EC matrix and exert activities including promoting proliferation, metastasis, angiogenesis and immunosuppression [24–26]. Fourth, platelets interact directly with tumor cells, releasing factors that promote tumor growth, invasion and angiogenesis [27]. Platelet can promote metastasis by stabilizing the retention of tumor cells in the vascular system, stimulating tumor cell proliferation and promoting tumor cell extravasation [28].

Based on the key evidence of trials in Western countries, NCCN guidelines recommend neoadjuvant therapy for locally advanced EC [29]. The rationale behind preoperative chemotherapy is twofold: (1) to downstage or downsize the primary tumor in order to ensure complete surgical resection and (2) to preemptively destroy any distant foci of micrometastatic disease [30]. The standard treatment for stage II/III EC is neoadjuvant therapy before RS, while surgery is the standard treatment for stage I EC [31]. However, in some patients, the disease may be highly malignant and may recur soon after surgery. Many studies have shown that postoperative adjuvant therapy has survival benefits for patients with T3-T4 stage EC, positive lymph node metastasis and positive margins [32]. Consistent with the observation results, this study found that receiving neoadjuvant and postoperative combined therapy were independent prognostic factors. Regarding nerve and vascular invasion, nerve invasion is a common phenomenon in a variety of cancers [33, 34], and nerves innervating the esophagus provide a favorable microenvironment that can promote tumor cell proliferation and spread, thereby worsening patient prognosis. Vascular invasion is an vital step in cancer metastasis and a major cause of cancer morbidity and mortality. The detection of vascular invasion in primary tumors is a marker of metastatic potential [35]. Consistent with the observations, the present study indicated that either neoadjuvant therapy or postoperative combination therapy, and the lack of nerve and vascular invasion were significantly associated with a favorable outcome.

A visual medical nomogram is an advanced prognostic model that can individually predict patient prognosis by integrating multiple clinicopathological factors. At present, nomograms have been developed for a variety of malignant tumors, and their predictive value is greater than that of the traditional TNM staging system. Some experts suggest taking it as an alternative or even a new standard [36]. Since both univariate and multivariate analyses of this study suggest that LMR and AISI are independent risk factors for the long-term survival of patients with EC after RS, we included LMR, AISI, statistically significant clinicopathological parameters and peripheral blood count characteristics in the nomogram. The aim was to individually predict the PFS and OS rates of postoperative patients with EC. In addition, the calibration curves showed a high consistency between the prognostic nomogram predicting the 1-, 3-, and 5 year survival rates and the actual observed values. If further validation can be completed in multicenter, large-scale trials and prospective studies, our nomograms may help clinicians predict the prognosis of EC.

This study has several limitations. First, due to the single-center retrospective design, selection bias was inevitable, and external validity was limited. We obtained some information on postoperative treatment and survival outcomes during follow-up through telephone conversations with the patients or their relatives, which may induce recall bias. Second, although the current study adopts strict inclusion and exclusion criteria, serum markers may be affected by other conditions, so the results should be treated cautiously. Third, in our study, the median OS for EC patients was only 25.600 months, significantly lower than the 100.100 months previously reported in Chinese randomized trials [37]. The possible reasons are as follows: first, the main follow-up endpoint of our study is five years; however, in previous Chinese randomized trials, the longest follow-up time can be more than 10 years; second, in our study, we also included patients with TNM stage III + IV, and patients with advanced stage may have a poor prognosis. Fourth, to understand the relationship between cancer patients and inflammation due to the long course of the disease, it is necessary to continuously monitor blood cell-derived indicators and other inflammatory indicators in future studies. Fifth, in this study, the cutoff values of the inflammatory indicators were calculated according to the highest Youden index of the ROC curves. The best cutoff values of these indicators were also calculated through RCS analyses. However, according to the results, the cutoff values obtained by the two methods were not exactly the same. Nevertheless, to date, there are no clear cutoff values for determining the prognosis of EC patients [38]. Therefore, in the future, we plan to explore the best cutoff values and the underlying molecular mechanisms.

## Conclusion

LMR and AISI, which are easy to obtain and suitable for clinical application, can be used as independent influencing factors to evaluate the inflammatory state and prognosis of patients with EC. In addition, nomograms have high clinical application value. They can intuitively predict the prognosis of patients with EC and help clinicians formulate or adjust reasonable diagnosis and treatment plans in a timely manner.

## Data Availability

The datasets used and/or analyzed during the current study are available from the corresponding author on reasonable request.

## Abbreviations

EC: Esophageal cancer
ESCC: Esophageal squamous cell carcinoma
RS: Radical surgery
LASSO: Least absolute shrinkage and selection operator
C-index: Index of concordance
ROC: Receiver operating characteristic
AUC: Area under the curve
DCA: Decision curve analysis
IDI: Integrated discrimination improvement
NRI: Net reclassification improvement
AJCC: American joint committee on cancer
RCS: Restricted cubic spline
OS: Overall survival
AISI: Aggregate index of systemic inflammation
RLR: Red blood cell distribution width-to-lymphocyte ratio
PFS: Progression-free survival
TNM: Tumor-node-metastasis
NLR: Neutrophil-to-lymphocyte ratio
PLR: Platelet-to-lymphocyte ratio
LMR: Lymphocyte-to-monocyte ratio
SII: Systemic immune-inflammation index
NMR: Neutrophil-to-monocyte ratio
NLPR: Neutrophil/(lymphocyte × platelet ratio)
SIRI: Systemic inflammation response index
CT: Computed tomography
HR: Hazard ratio
CI: Confidence interval
ANOVA: Analysis of variance
AIC: Akaike information criterion
TAMs: Tumor-associated macrophages
NCCN: National comprehensive cancer network
RDW: Red blood cell distribution width
Hb: Hemoglobin
WBC: White blood cell
Neut: Neutrophil
Mono: Monocyte
ALB: Albumin
PA: Prealbumin
LDL: Low density lipoprotein
CPR: C-reactive protein
